# A mathematical model of quorum sensing regulated EPS production in biofilm communities

**DOI:** 10.1186/1742-4682-8-8

**Published:** 2011-04-10

**Authors:** Mallory R Frederick, Christina Kuttler, Burkhard A Hense, Hermann J Eberl

**Affiliations:** 1 Department of Mathematics and Statistics, University of Guelph, 50 Stone Rd E, Guelph ON Canada N1G 2W1; 2 Center of Mathematical Sciences, Technical University of Munich, Boltzmannstr. 3, 85748 Garching, Germany; 3 Institute of Biomathematics and Biometry, HelmholtzCenter Munich, Ingolstädter Landstr. 1, 85764 Neuherberg, Germany

## Abstract

**Background:**

Biofilms are microbial communities encased in a layer of extracellular polymeric substances (EPS). The EPS matrix provides several functional purposes for the biofilm, such as protecting bacteria from environmental stresses, and providing mechanical stability. Quorum sensing is a cell-cell communication mechanism used by several bacterial taxa to coordinate gene expression and behaviour in groups, based on population densities.

**Model:**

We mathematically model quorum sensing and EPS production in a growing biofilm under various environmental conditions, to study how a developing biofilm impacts quorum sensing, and conversely, how a biofilm is affected by quorum sensing-regulated EPS production. We investigate circumstances when using quorum-sensing regulated EPS production is a beneficial strategy for biofilm cells.

**Results:**

We find that biofilms that use quorum sensing to induce increased EPS production do not obtain the high cell populations of low-EPS producers, but can rapidly increase their volume to parallel high-EPS producers. Quorum sensing-induced EPS production allows a biofilm to switch behaviours, from a colonization mode (with an optimized growth rate), to a protection mode.

**Conclusions:**

A biofilm will benefit from using quorum sensing-induced EPS production if bacteria cells have the objective of acquiring a thick, protective layer of EPS, or if they wish to clog their environment with biomass as a means of securing nutrient supply and outcompeting other colonies in the channel, of their own or a different species.

## Background

### Biofilms, quorum sensing, and EPS

Biofilms are microbial communities encased in a layer of extracellular polymeric substances (EPS), adhered to biotic or abiotic surfaces. Bacteria preferentially reside in biofilms, rather than in isolation as planktonic cells. In a biofilm, bacteria are protected by the EPS matrix from external stresses, and carry out a wide range of reactions which are relevant in many disciplines, such as environmental engineering, food processing, and medicine [1].

Quorum sensing is generally interpreted as a cell-cell communication mechanism used by several bacterial taxa to coordinate gene expression and behaviour in groups, based on population densities [2]. Initially, bacteria cells produce and release low amounts of signalling molecules, called autoinducers (e.g., acyl-homoserine lactones (AHL) in Gram-negative bacteria). Concurrently, the cells measure the environmental concentration of the autoinducer. When a critical concentration is reached, changes in gene expressions are induced. In most bacterial autoinducer systems, the autoinducer synthase gene itself is upregulated, initiating positive feedback, and the bacteria subsequently produce AHL molecules at an increased rate. As a number of traits in bacterial biofilms relevant for human and plant health are regulated via autoinducers [3,4], a comprehensive understanding of quorum sensing systems is highly desirable. EPS is composed of organic molecules such as polysaccharides, proteins, and lipids. The EPS matrix provides several functional purposes for the biofilm, such as protecting bacteria from environmental threats, providing mechanical stability, and degrading macromolecules to be used by the cells [5]. EPS is thought to indirectly store nutrients, which could later be converted to an available form and used as an energy source during periods of low nutrient availability [6-9].

### Modelling of biofilms and quorum sensing

Biofilms are complex systems that can be viewed simultaneously as microbial ecological communities and as mechanical objects. Traditional one-dimensional biofilm models were formulated as free boundary value problems of semi-linear diffusion reaction systems (see [10]). Newer models take the spatially heterogeneous structure of biofilms into account and are formulated as spatially multi-dimensional models. A host of mathematical modelling techniques has been proposed to model biofilms, including stochastic individual based models, stochastic cellular automata models, and a variety of deterministic partial differential equation models. Some examples for such approaches are: [11-25]. These models of biofilm structure are usually coupled with diffusion-reaction models for growth controlling substrates such as nutrients and oxygen. This leads to hybrid models which are mathematically difficult to analyse and often only amendable to computational simulations. In most biofilm models, EPS is not explicitly included but implicitly subsumed in the variables that describe biomass and biofilm structure. Some early exceptions are the one-dimensional model of [26], the hybrid individual-continuum model of [11], the hydrogel model of [20], and the diffusion-reaction model [27].

For our study we build on the prototype biofilm model of [16], in which the biofilm structure is described by a determinstic, density-dependent diffusion-reaction equation with two nonlinear diffusion effects: porous medium degeneracy and a super-diffusion singularity. This model has been extended to explicitly account for EPS in [27] based on [26], and to model quorum sensing in [28]. In the current study, we combine both effects.

Although the various multi-dimensional biofilm models are based on fundamentally different assumptions, such as ecological vs. mechanical properties of biofilms, and although they utilise different mathematical concepts, such as discrete stochastic vs deterministic continuous descriptions, they have been shown to predict similar biofilm structures in [10]. More recently it was formally shown that the prototype density-dependent diffusion-reaction biofilm model, on which our study is based, can be derived from a spatially discrete lattice model that is related to cellular automata biofilm models [29]. In [28], it was also shown that the same prototype density-dependent diffusion-reaction model can likewise be derived from a the same hydrodynamic description of biofilms that underlies the biofilm model introduced by [15]. Thus, the density-dependent diffusion model of biofilms can be understood as bridge between ecological and continuum mechanical views in biofilm modelling. The idea of using nonlinear diffusion processes, in the form of modified Cahn-Hilliard equations, to describe the propagation of the biofilm/water interface, is also used in current, physically more involved phase field models, as introduced in [24].

Initial mathematical models of quorum sensing describe the phenomena in suspended bacteria cultures [30-32]. These models focus on predicting the rapid switch in proportions of down- and upregulated sub-populations of bacteria in a batch culture, which is the characteristic positive-feedback feature of quorum sensing systems. Papers [33-35] extended the work of early models to study quorum sensing in a growing biofilm, identifying key physical kinetics parameters required for induction. More recent models describe growth in two dimensions [28], and include the effects of hydrodynamics [28,36,37]. A variety of applications motivate development of specific quorum sensing and biofilm models. For example, papers [34,35] determine the critical depth the biofilm must grow to, as a function of pH, in order for induction to occur. The models of [38-40] detail biochemical pathways in quorum sensing systems, also describing anti-quorum sensing treatments for applications in the medical field. The role of convective and diffusive transport of signal molecules in inter-colony communication within biofilm communities is investigated in [28].

These models share a common element: autoinducer molecules (e.g., AHL) are produced by downregulated bacteria, and AHL production is greatly enhanced when the characteristic *switch *(change from low to high quorum sensing activity) rapidly occurs throughout the biofilm.

Much mathematical modelling research has been conducted to understand when biofilms partake in quorum sensing activity, for example, determining population thresholds [30,31], critical biofilm depth [34,35], and the influence of the hydrodynamic and nutritional environment [28,36,41]. There have, however, been few studies that look at the reverse effect - the effect of quorum sensing induction on biofilms. Once biofilm cells are upregulated, AHL is produced at an increased rate, but the question of whether the biofilm behaves differently, grows differently, or undergoes some other functional change, remains largely unanswered.

We expand on the works of [38-40,42]. Study [42] analyzes the effectiveness of the modelled anti-quorum sensing therapies by comparing growth rates of the biofilms, and states that quorum sensing activity may be detected by EPS production and associated enhanced biofilm growth. Based on the findings of [43], it is assumed in [42] that EPS production is regulated by quorum sensing, and models significantly enhanced EPS production by upregulated cells. With our model, we will study in detail how the process of quorum sensing-regulated EPS production impacts biofilm growth and development in a two-dimensional patchy biofilm community with slow background flow, under various environmental conditions. Our objective is to understand the relationship between quorum sensing, biofilm growth, and EPS production, and investigate the benefits a biofilm receives by using quorum sensing-regulated EPS production.

To validate the claim that quorum sensing controls EPS production, and to what degree, we turn to the experimental literature. In many studies, quorum sensing has been found to impact the quality of EPS. For example, study [44] showed differences in biofilm appearance with and without expression of the *pelA *gene, which is essential for the production of the EPS matrix. In a later study of the quorum sensing-regulated expression of the *PelA *enzyme, it was shown that *pel*-genes are required for EPS production [45]. On the other hand, [46] found many factors which affect the quality of the EPS matrix to be regulated by quorum sensing in the early development stage, such as channel production within the biofilm, swarming activity, and lipid production. Also, many studies have shown the connection between quorum sensing and mucosity [47-49]. Quorum sensing regulates components of EPS (e.g., EPS II, polysaccharides) which contribute to the mucosity, thus impacting the biofilm matrix. These studies support the idea that the amount of EPS production per cell might be influenced by quorum sensing, but do not show to what degree.

There are some examples of bacteria species, mostly plant pathogens, in which a quantitative increase of EPS production by quorum sensing regulation has been demonstrated. In [50], quorum sensing was found to regulate alginate production in *Pseudomonas syringae*. Alginate is an important component of EPS, and without quorum sensing, alginate levels were 70% lower. However, the impact on biofilm thickness is not described, so conclusions cannot be drawn regarding whether overall EPS is significantly reduced by the drop in alginate levels.

In [51] it is concluded that the amount of EPS production per cell in a *Pantoea stewartii *biofilm is increased by quorum sensing, though the degree of production is not given. Similarly, in [52] is claimed that quorum sensing upregulated EPS production in the plant pathogen *Erwinia amylovora*, but do not provide quantitative data. However, images are shown, from which the upregulated EPS may be estimated as a factor five to ten increase. This is supported by the experiments in [53], in which an approximately ten-fold increase of EPS production in a *Pantoea stewartii *biofilm upon QS induction was discovered. Though many studies have established connections between quorum sensing activity and qualitative changes in EPS or other structural components, there are very few quantitative studies which investigate the amount of EPS produced through quorum sensing regulation. We choose to use the direct values for change in EPS production as reported in [53] as an estimate for the difference in downregulated and upregulated cell production rates in our system.

### Aim of study

In previous research, we developed a two-dimensional model of quorum sensing in patchy biofilm communities in an early development stage to study how the hydrodynamic environment and nutrient conditions contribute to biofilm growth, spatiotemporal quorum sensing induction patterns, and flow-facilitated intercolony communication [28].

In this paper, we will extend this model to include a response from the biofilm once quorum sensing has been induced. The upregulated cells not only produce AHL at increased rates, but produce EPS at an increased rate as well. We wish to investigate whether QS regulated EPS production provides a benefit (in some sense) over a EPS production strategy at fixed rate. In order to do so, we address two main research questions with our model:

1. *How does quorum sensing-regulated EPS production impact the growing biofilm?*

2. *Why is it beneficial for the biofilm to regulate EPS production using a quorum sensing mechanism?*

Answers to these questions will be sought through numerical experiments that simulate the growth of biofilms in microfluidic chambers.

## Mathematical Model and Simulation Design

### Model assumptions

We formulate a mathematical model that describes quorum sensing in a growing biofilm community in a narrow conduit which consists of several colonies, mimicking conditions that occur in soil pores or plant/blood vessels. The biofilm is assumed to consist of bacterial cells and EPS, and it is described by the local densities of its constituents. The biofilm proper is the region in which these densities are not zero; it is surrounded by the bulk liquid. The biofilm expands due to cell growth and EPS production, both of which are coupled to the availability of a carbon nutrient. The nutrient is assumed to be dissolved. In the aqueous phase surrounding the biofilm, the nutrient is transported by bulk flow convection and by Fickian diffusion. In the biofilm itself it diffuses, although at reduced rate due to the increased diffusive resistance of the EPS and cells. Nutrients are degraded in the biofilm by the growing cells for growth and EPS production.

We distinguish between down- and upregulated bacterial cells. Upregulation and downregulation are controlled by the local concentration of AHL. Upregulation occurs locally when and where the AHL concentration exceeds a threshold. If the AHL concentration in a (partially) upregulated biofilm colony drops below this critical threshold, the upregulated cells become downregulated. AHL is also assumed to be dissolved. AHL is transported by convection and diffusion in the surrounding aqueous phase, and by diffusion in the biofilm, also at a reduced rate. After AHL is produced by the bacteria, it diffuses into the aqueous phase. Upregulated cells produce AHL at a higher rate (by one order of magnitude) than downregulated cells, and decay abiotically, at a rate much slower than they are produced.

We assume that up- and downregulated cells grow at the same rate, but upregulated cells produce EPS at much higher rates (tenfold). Moreover, we assume that the average cell size for down- and upregulated bacteria is the same, i.e., the maximum cell and EPS density is the same for both cell types. The increased production of EPS implies an increased nutrient consumption of upregulated cells. Based on the parameters for EPS production kinetics and stoichiometry of [26], we estimate with a simple rule of proportions that upregulated cells consume approximately twice the amount of nutrients that down-regulated cells consume. We do not distinguish between the EPS that is produced by each type of bacteria, but combine them into one EPS fraction.

In addition to bacteria that engage in quorum sensing, i.e., switch between down- and upregulated states, we also consider non-quorum sensing bacteria species, which behave as either downregulated or upregulated cells, in regards to parameters for growth, consumption, and EPS production. These non-quorum sensing cells carry an AHL-receptor mutation and cannot be upregulated or produce any AHL. Although they are technically mutant cells, we will refer to these non-quorum sensing bacteria as different species throughout the paper.

We formulate this model in the framework of the density-dependent nonlinear diffusion model for biofilms which was originally introduced for a prototype single species biofilm in [16], and has since been extended to multi-species systems. Quorum sensing was first included in this model in our earlier study [28]. In the current study, we expand on this model by explicitly accounting for EPS, which was previously implicitly subsumed in the biomass fractions. Our model of EPS production is based on the one-dimensional biofilm model of [26]. Some authors suggest that under conditions of severe nutrient limitations, EPS could be broken down and converted into nutrients by the cells [6-9,54]. Following [26], we include this process as an option in our model and investigate whether it affects quorum sensing activity and biofilm composition.

### Governing equations

The mathematical model for biofilm growth, quorum sensing, and EPS production, based on the above assumptions, is formulated as a differential mass balance for the bacterial biomass fraction, EPS, growth-promoting nutrient substrate and quorum sensing molecules.

Following the usual convention of biofilm modelling, the density of the particulate substances (bacterial cells and EPS), is expressed in terms of the volume fraction that they occupy [10]. We denote the volume fraction locally occupied by downregulated quorum sensing cells by *M*_0 _[-], the volume fraction of upregulated quorum sensing cells by *M*_1 _[-]. Their densities are accordingly *M*_0 _*M_max _and *M*_1_*M_max_, where the constant *M_max _*[*gm*^-3^] is the maximum biomass density, in terms of mass COD per unit volume.

The non-quorum sensing bacteria are accordingly expressed in terms of the volume fractions *M*_2 _(downregulated cells) and *M*_3 _(upregulated cells). A summary of the cell types and behaviours is given in Table 1.

**Table 1:** Summary of the cell types and functions used in the model.

Cell Type	Description
*M*_0_	downregulated QS, low EPS producer
*M*_1_	upregulated QS, high EPS producer
*M*_2_	non-QS, low EPS producer
*M*_3_	non-QS, high EPS producer

Cells are classified as quorum sensing (QS) or non-quorum sensing (non-QS), and low or high EPS producers.

Similarly, EPS density is expressed in terms of its variable volume fraction *EPS *[-] and the constant maximum EPS density *EPS_max _*[*gm*^-3^], as EPS * *EPS_max_*.

The dissolved growth controlling nutrient substrates and the dissolved quorum sensing molecules are described in terms of their concentrations *C *[*gm*^-3^] and *AHL *[*nM*].

The differential mass balances for the dependent variables *M*_0,1,2,3_, *EPS*, *C*, *AHL *are obtained as:(1)(2)(3)(4)(5)(6)(7)

where in the mass balances for the particulate substances, the constant densities *M_max _*and *EPS_max _*cancelled. The total volume fraction occupied by the biofilm is denoted by *M*, where

The two-dimensional computational domain Ω consists of a liquid phase with no biomass, Ω_1_(*t*) = {(*x, y*) ∈ Ω : *M*(*t*, *x*, *y*) = 0}, and the solid biofilm phase, Ω_2_(*t*) = {(*x*, *y*) ∈ Ω : *M*(*t*, *x*, *y*) > 0}.

These regions change as the biofilm grows.

The diffusion coefficient for the biomass fractions (*D_M _*(*M*)) is density dependent, and is formulated according to [16] as

The diffusion coefficient can be assumed to be the same for all bacterial fractions and the EPS because we do not distinguish the cells with respect to size and growth behaviour, and because EPS and cells diffuse together. The biomass motility coefficient *d_m _*[*m*^2^*d*^-1^] is positive but much smaller than the diffusion coefficients of the dissolved substrates. Exponents *a *> 1 [-] and *b *> 1 [-] ensure biofilm expansion when *M *approaches 1 (implying all available space is filled by biomass), and little or no expansion provided *M *is small. This choice of diffusion coefficient ensures a separation of the biofilm and its surrounding aqueous phase, and that the maximum cell density will not be exceeded. The latter effect is of the type of a superdiffusion singularity, the former of the type of the porous medium equation degeneracy.

The diffusion coefficients for *C *and *AHL *are lower in the biofilm than in the surrounding aqueous phase [55]. We let

where *D_C_*(0) and *D_AHL_*(0) are the diffusion coefficients in water, and *D_C_*(1) and *D_AHL_*(1) are the diffusion coefficients in a fully developed biofilm [*m*^2^*d*^-1^]. Although these diffusion coefficients depend on the biomass density as well, they do so in a non critical way. Since *D_C, AHL_*(0) and *D_C, AHL_*(1) are positive constants within one order magnitude, substrate diffusion is essentially Fickian.

The model includes diffusive transport of carbon substrate and AHL in the biofilm, and both convective and diffusive transport in the surrounding aqueous phase of the biofilm. The convective contribution to transport of *C *and *AHL *in the aqueous phase is controlled by the flow velocity vector *w *= (*u*, *v*), where *u *and *v *[*md*^-1^] are the flow velocities in the *x*- and *y*- directions. The flow in the aqueous phase is described by a thin-film approximation to the incompressible Navier-Stokes equations [56]. In order to drive the flow in the channel, we specify the volumetric flow rate in terms of the non-dimensional Reynolds number *Re*. The growth and decay processes incorporated into our model are:

• growth of bacterial cells, controlled by the local availability of carbon substrate, in equations (1)-(4): the maximum specific growth rate is denoted by *κ*_3 _[*d*^-1^], dependency on *C *is described by standard Monod kinetics where *κ*_2 _[*gm*^-3^] is the half saturation concentration.

• natural cell death, at rate *κ*_4 _[*d*^-1^], in equations (1)-(4),

• upregulation of downregulated biomass, i.e. the conversion of *M*_0 _cells into *M*_1 _cells in equations (1) and (2), as a consequence of AHL concentration inducing a change in gene expression, and a constant rate of back-conversion. The parameter *κ*_5 _[*d*^-1^*nM *^-*n*^] is the quorum sensing regulation rate -- the rate at which downregulated bacteria become upregulated, and vice versa. *τ *[nM] is the threshold AHL concentration locally required for quorum sensing induction to occur. The coefficient *n *(*n *> 1) describes the degree of polymerisation in the synthesis of AHL. We model the dimerisation process for AHL, assuming that dimers of receptor-AHL complexes are necessary for the transcription of the AHL-synthase gene. Assuming mass action law kinetics, this process gives *n *= 2, however, the value of *n *used here is slightly higher, as further synergistic effects are lumped into this parameter as well [28].

• production of EPS by the bacterial cells at rates proportional to the bacterial growth rates, in equation (7): the EPS production rate is

in [*d*^-1^] where the yield coefficients *Y_i_*(*EPS*) [-] describe the amount of EPS produced per unit bacterial biomass of type *M*_0,1,2,3_.

• nutrient consumption by bacterial biomass in (5): the maximum specific substrate consumption rates are denoted by

in [*gm*^-3^*d*^-1^], where *M_max _*is the maximum cell density, and *Y_i _*[-] are the yield coefficients that incorporate both, the amount of nutrient required for biomass growth and for EPS production

• abiotic AHL decay, at rate *σ *[*d*^-1^], in equation (6)

• AHL production by both quorum-sensing cell types *M*_0 _and *M*_1 _in (6) at different rates: the AHL production rate of downregulated quorum sensing bacteria is *α *[*nM*/(*gm*^-3^*d*^-1^)], and the increased production rate of upregulated quorum sensing bacteria is *α *+ *β *[*nM d*^-1^]

• when carbon becomes limited, EPS may be used as a food source, in equations (5) and (7). This process is represented by an inhibition term, in which EPS is transformed into carbon at rate *δ *[*d*^-1^], with inhibition constant *κ*_6 _[*gm*^-3^]; the rate  in (5) is related to *δ *by a yield coefficient and a constant conversion factor, see [26]; to neglect the EPS consumption process, we let *δ *= 0 and .

For the numerical treatment, the above model is non-dimensionalized with the choices:

where *L *is the flow channel length, and  is the characteristic time scale for biomass growth. The new dimensionless concentration variables are:

where *C_bulk _*is the bulk substrate concentration (the amount of substrate *C *supplied at the inflow boundary). Note that the volume fractions *M_i_*, *i *= 0, ..., 3 and *EPS *were originally defined as dimensionless variables. The new reaction parameters are:

The dimensionless diffusion coefficients become:

The non-dimensionalized equations are then:

The parameters used in our simulations and their non-dimensional values are listed in Table 2. The biofilm growth parameters, the EPS production parameters, and the substrate diffusion coefficients were chosen from the range of standard values in biofilm modelling literature [10,26], and the biomass diffusion coefficient values (*d_M_*, *a*, *b*) were selected from [56]. The quorum sensing parameters *κ*_5_, *α*, *β*, *γ *and *n *were derived from experiments on the kinetics of suspended *P. putida *IsoF cultures and the AHL molecule 3-oxo-C10-HSL [57]. In experimental quorum sensing literature, the threshold AHL concentration required for induction, *τ*, ranges from less than 5 nM to above 200 nM. Following [58], we have chosen the relatively low value of *τ *= 10 nmol/L to allow for induction to occur at an early stage of biofilm growth. We have selected these parameters in order to analyze the general behaviour of a system of biofilms and quorum sensing, i.e., the analysis is not specific to *P. putida *and AHL. The flow velocity is Re = 10^-4^, which is well within the creeping flow regime. At this low flow rate, the dimensionless Peclet number, which estimates the relative contributions of convective and diffusive mass transport, is *Pe *≈ 1.0, indicating that the system is neither convection- nor diffusion-dominated. In particular, in convection dominated cases (*Pe *>> 1), it has been shown that AHL can be washed out without contributing to up-regulation [28,37]. Moreover, following [56], biofilm deformation and shear induced detachment can be neglected at these low flow velocities.

**Table 2:** Model parameters in the high nutrient case.

Parameter	Description	Source	Value
*κ*_10_	Rate of *C *consumption by *M*_0_	W	923
*κ*_11_	Rate of *C *consumption by *M*_1_	W	1846
*κ*_12_	Rate of *C *consumption by *M*_2_	W	923
*κ*_13_	Rate of *C *consumption by *M*_3_	W	1846
*κ*_2_	Monod half sat. const.	W	0.02
*κ*_3_	Max specific growth rate of bacteria	H	1.0
*κ*_4_	Bacterial lysis rate	W	0.2083
*κ*_5_	Quorum sensing upregulation rate	F	2.5
*κ*_6_	Monod half sat. const.	H	0.04
*δ*	EPS conversion to C rate (C equation), if included	H	0.28
	EPS conversion to C rate (EPS equation), if included	H	11.2
*σ*	Abiotic degradation rate of AHL	F	0.1109
*α*	Constitutive production rate of AHL	F	920
*β*	Induced production rate of AHL	F	9200
*n*	Degree of polymerisation	F	2.5
*γ*_0_	*M*_0 _EPS production rate	H	0.84
*γ*_1_	*M*_1 _EPS production rate	H	8.4
*γ*_2_	*M*_2 _EPS production rate	H	0.84
*γ*_3_	*M*_3 _EPS production rate	H	8.4
*M_max_*	Maximum cell density	H	24·10^3^
*EPS_max_*	Maximum EPS density	H	4·10^3^
*D_C _*(0), (1)	Substrate diffusion coefficients	ES	0.67, 0.54
*D_AHL_*(0), (1)	AHL diffusion coefficients	HR	0.52, 0.26
*a*	Diffusion coefficient parameter	ES	4.0
*b*	Diffusion coefficient parameter	ES	4.0
*d_M_*	Biomass motility coefficient	ES	6.67e-09
*H/L*	Channel aspect ratio	ES	0.1

References: ES = [56], H = [26], HR = [64], F = [57], W = [10]

### Computational approach

The numerical solution of the density-dependent diffusion-reaction model is computed using a semi-implicit finite difference-based finite volume scheme, formulated for the concentrations in the centers of the grid cells. Time integration uses a non-local (semi-implicit) discretization in the fashion of non-standard finite difference methods. The time-step size is variable and chosen in order to ensure stability, positivity, boundedness (by 1), and a finite speed of interface propagation [59]. In our application, the computational domain is discretized on a uniform rectangular grid of size 2000 × 200.

In each time step six sparse, banded diagonal linear algebraic systems (one for each of *M*_0_, *M*_1_, *M*_2/3_, *C*, *AHL*, and *EPS*) are solved with the stabilized biconjugate gradient method. The flow field is calculated using the analytical approximation of [56].

The numerical method was first introduced for single-species biofilms in [59] and then extended to biofilm systems with several microbial species in [60] and [61], the latter also containing a stability analysis. A computational convergence study can be found in [62]. These results carry over qualitatively to the study at hand. The method is implemented in OpenMP for execution on multi-core and shared memory multi-processor architectures; the parallelization behaviour is documented in [61]. The simulations were conducted on an Intel Itanium based SGI Altix 450, typically using 12 cores concurrently.

### Simulation setup

Three different types of biofilms will be studied: quorum sensing (*M*_0_, *M*_1 _cells only), non-quorum sensing (*M*_2 _or *M*_3 _cells only), and mixed (*M*_0_, *M*_1_, and one of *M*_2 _or *M*_3 _cells). Two nutrient conditions are tested: high and low (differing by a factor of two), and simulations are performed with and without the biological process of EPS consumption; the parameters *δ *and  are set equal to zero when EPS consumption is excluded. A summary of the simulation experiments is given in Table 3. Our simulations will give us qualitative information about quorum sensing and biofilm systems. Numerical results, including time, are described using non-dimensional measures, and should not be deemed as quantitative conclusions.

**Table 3:** Summary of the simulation experiments.

Biofilm Name	Biofilm Type	Nutrient Case	EPS consumption
QS	*M*_0_, *M*_1_	high, low	yes, no
*M*_2 _non-QS	*M*_2_	high, low	yes, no
*M*_3 _non-QS	*M*_3_	high, low	yes, no
*M*_2 _mixed	*M*_0_, *M*_1_, *M*_2_	high	yes, no
*M*_3 _mixed	*M*_0_, *M*_1_, *M*_3_	high	yes, no

Our biofilm model is on a mesoscopic scale, and so the computational domain is considered to be a small portion, or open subdomain, existing within a larger reactor. The boundary conditions we choose describe both the reactor type and the operating conditions in which the experiment is conducted, and connect the computational domain to the outside physical environment. Our computational domain is representative of a microfluidics chamber which receives fluid at the left (inflow) boundary from a large, well mixed reactor. Carbon is supplied into the channel from the upstream boundary, but no AHL may enter into the flow channel from upstream. AHL and carbon in the dissolved liquid phase Ω_1 _may exit the system via convective transport.

Specifically, the following boundary conditions are imposed on our domain Ω = [0, *L*]×[0, *H*]:

• For *M*_0_, *M*_1_, *M*_2_, *M*_3 _and *EPS*, no flux conditions everywhere (n is the direction of the outward normal): *∂_n_M*_0 _= 0, *∂_n_M*_1 _= 0, *∂_n_M*_2 _= 0 *∂_n _M*_3 _= 0, *∂_n_EPS *= 0 on *∂*Ω

• For *C *and *AHL*, no diffusive flux conditions everywhere except for on inflow, where we specify the bulk concentration: *C *= 1, *A *= 0 for *x *= 0, *∂_n_C *= 0, *∂_n _A *= 0 everywhere else.

The initial conditions used are:

• An inoculation of the bottom surface of the channel with 16 colonies, each with a density of 0.3. Biofilm colonies are placed randomly along the channel, at an offset from the channel entrance and exit, to avoid unphysical boundary effects. This random placement mimics experimental difficulties in controlling where bacteria settle. The type of cell inoculated depends on the biofilm being grown: either quorum sensing (16 *M*_0 _cells), non-quorum sensing (16 *M*_2 _or *M*_3_), or mixed (8 *M*_0 _and 8 *M*_2_, or 8 *M*_0 _and 8 *M*_3_)

• AHL = 0, EPS = 0; initially, biomass consists of cells only, but EPS and AHL production begins immediately upon the start of the simulation

• C = 1.

The simulations finish when an imposed stopping criterion is met: the biofilm height reaches 80% of the channel height. This ensures the simulation stops before clogging effects take place; when the biofilm height approaches the top of the channel, local flow velocities and shear forces increase to the level that detachment processes would no longer be negligible, leading to a breakdown of the biofilm growth model.

### Analysis

To interpret the results of computer simulations of our model, we will provide two-dimensional visualizations of the simulations, and use the following quantitative measures. The volume fraction of the domain occupied by the biofilm (cells and EPS), or the occupancy, is a simple measure of biofilm size. The occupancy is averaged over the whole regarded volume:

The total downregulated quorum sensing cell biomass in the system, *M*_0*total*_, is the volume fraction of *M*_0 _multiplied by maximum cell density:

The total *M*_1_, *M*_2_, and *M*_3 _cell biomasses, and the EPS biomass, are computed similarly. The total biomass is therefore:

The occupancy and total cell and EPS biomass measures will be used to compare the growth and composition of the biofilm over time.

We will use the following abbreviations: quorum sensing (QS), non-quorum sensing (non-QS).

## Results

The results of the simulation experiments summarized in Table 3 will be described in the following sequence:

• *Example simulation of QS controlled EPS production in a biofilm*: an example simulation of a quorum sensing biofilm under high nutrient conditions.

• *Simulations without the EPS consumption process*: simulations of biofilms that do not include the process of EPS consumption. First, QS and *M*_2 _and *M*_3 _non-QS biofilms are compared under high and low nutrient conditions. Second, *M*_2 _and *M*_3 _mixed biofilms are regarded.

• *Simulations with the EPS consumption process*: the experiments of the previous section are repeated, but the process of EPS consumption is included. QS and *M*_2_, *M*_3 _non-QS biofilms under high and low nutrient conditions are described first, followed by *M*_2 _and *M*_3 _mixed biofilms.

• *Effect of random colony placement in mixed biofilms*: a discussion on the effects of random initial colony placement in *M*_2 _and *M*_3 _mixed biofilms on quorum sensing induction.

### Example simulation of QS controlled EPS production in a biofilm

To simulate growth of a QS biofilm, the bottom surface of the channel is inoculated with sixteen *M*_0 _colonies. A high supply of substrate enters the channel from the inflow boundary, and the process of EPS consumption is neglected.

**Figure 1:**
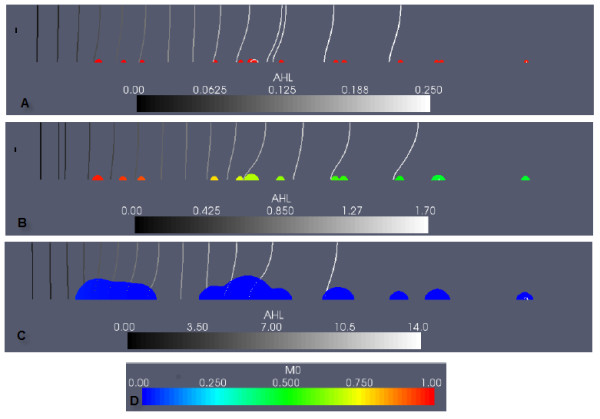
**Example simulation: QS biofilm in the case of high nutrient conditions, and no EPS consumption**. The colour scale (E) in the subfigures represents the fraction of downregulated cells in the biofilm (*M*_0_/(*M*_0 _+ *M*_1_)). AHL concentration lines are shown in black and white, equidistantly distributed between zero and the maximum AHL value at each time. A non-dimensional value of AHL = 1.0 is required for upregulation. (A) shows the QS biofilm, before induction occurs (t = 5.0, max AHL = 0.44). In (B), the downstream colonies have upregulated (t = 6.0, max AHL = 1.71). (C) shows the biofilm after induction has occurred (t = 8.0, max AHL = 14.1).

The growth period begins with biomass in the inoculated colonies growing and spatially spreading when the total biomass (*M*_0 _+ *M*_1 _+ *EPS*) locally approaches the maximum density, 1.0. In time, some neighbouring colonies begin to merge. Figure 1(a) depicts the biofilm before induction occurs; the colonies consist almost entirely of *M*_0 _cells.

AHL accumulates over time in the channel as it is produced by the growing colonies. Molecules produced by the colonies diffuse into the liquid region, and are transported downstream by convection and diffusion, causing AHL concentrations to increase in the main flow direction. The maximum AHL concentration found at the downstream boundary is a typical effect of flow facilitated convective transport [37]. In Figure 1(b), the switch to QS is occuring. Upregulation occurs locally when the non-dimensional AHL concentration reaches 1.0. Positive feedback in the quorum sensing system is then initiated -- upregulated cells produce AHL at ten times the downregulated rate, leading rapidly to large increases in AHL concentrations, and further upregulation of cells throughout the domain.

The downstream colonies begin to upregulate first, followed by the upstream colonies. This is an observation of flow-facilitated inter-colony communication -- AHL molecules produced by the large, upstream colonies are transported by convection and diffusion, contributing to upregulation in the smaller downstream colonies [28].

The biofilm in Figure 1(c) is fully upregulated, and EPS production has increased by a factor of ten. The biofilm grows and expands rapidly, until the flow channel becomes clogged with biomass and the maximum predetermined biofilm height is obtained.

**Figure 2:**
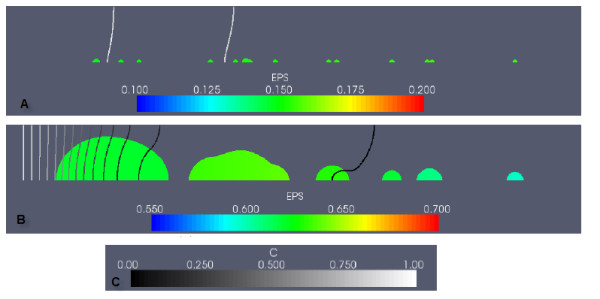
**Example simulation: QS biofilm in the case of high nutrient conditions, and no EPS consumption**. The colour scale in the subfigures represents the fraction of EPS biomass in the biofilm ((*EPS*)/(*M*_0 _+ *M*_1 _+ *EPS*)), showing mass composition of the biofilm. Carbon concentrations are normalized with respect to the value of the incoming carbon concentration (the non-dimensional parameter *C_bulk_*). (A) shows the QS biofilm at an early stage of growth, before induction (t = 5.0). (B) shows the biofilm after induction (t = 9.5). The carbon concentration colour scale is given in (C).

In Figure 2, the biofilm is shown again before and after induction, with the colour scale representing the proportion of cellular biomass (the fraction (*M*_0 _+ *M*_1_)/(*M*_0 _+ *M*_1 _+ *EPS*)), along with the concentration of the carbon nutrient *C*. Carbon concentrations decrease in the flow direction, due to consumption by biomass. In later timesteps, mid-channel and downstream colonies experience severe substrate limitations due to substrate consumption by the larger upstream colonies.

Prior to induction (Figure 2(a)), the biofilm composition by mass is approximately 15% EPS, 85% cells. Following induction (Figure 2(b)), EPS production rates are upregulated, resulting in a change in biofilm composition to 60-65% EPS. The large, merged, upstream colonies experienced the greatest increase in volume - in order for colonies to have increased growth due to upregulated EPS production rates, both upregulated cells and adequate nutrients are required. So although the downstream colonies are first to upregulate, these colonies lack the nutrients needed to expand quickly.

**Figure 3:**
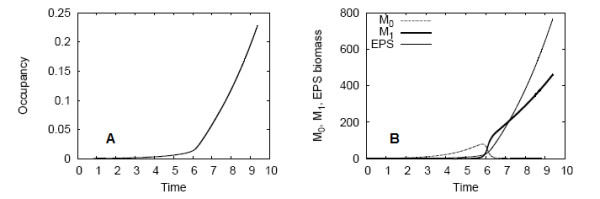
**Spatially averaged results for the example simulation**. The (A) occupancy and (B) *M*_0_, *M*_1_, and *M*_2 _biomass of the quorum sensing biofilm with high nutrient supply, excluding EPS consumption.

In Figure 3, the spatially averaged measures introduced in the "Analysis" Section are plotted, describing biofilm growth and EPS production in time. The biofilm occupancy (inclusive of cellular and EPS biomass) is plotted in Figure 3(a). The slope of the occupancy curve increases at *t *= 6.0, corresponding to induction, after which biofilm growth continues at an enhanced rate. The total *M*_0_, *M*_1_, and EPS biomass constituting the biofilm is shown in Figure 3(b). It is primarily the downregulated cell populations which grow in the initial time period, until induction occurs. Subsequently, *M*_0 _cells are rapidly upregulated to *M*_1 _cells, and the total *M*_0 _biomass in the biofilm declines while *M*_1 _cell representation increases. Upon completion of the simulation, the biofilm is fully upregulated. *M*_1 _produces EPS at the induced rate, so EPS biomass increases substantially after the switch as well.

### Simulations without the EPS consumption process

#### Quorum sensing and non-quorum sensing biofilms

In the first simulation experiment, QS biofilms (*M*_0_, *M*_1 _cells), the low-EPS producing *M*_2 _non-QS biofilms and high-EPS producing *M*_3 _non-QS biofilms were grown under high and low nutrient conditions, using the same initial distribution of colonies. Under the low nutrient condition, the concentration of incoming carbon (*C_bulk_*) is lowered, which affects the following non-dimensional parameters: *κ*_10 _= 1846, *κ*_11 _= 3692, *κ*_12 _= 1846, *κ*_13 _= 3692, *κ*_2 _= 0.04, .

**Figure 4:**
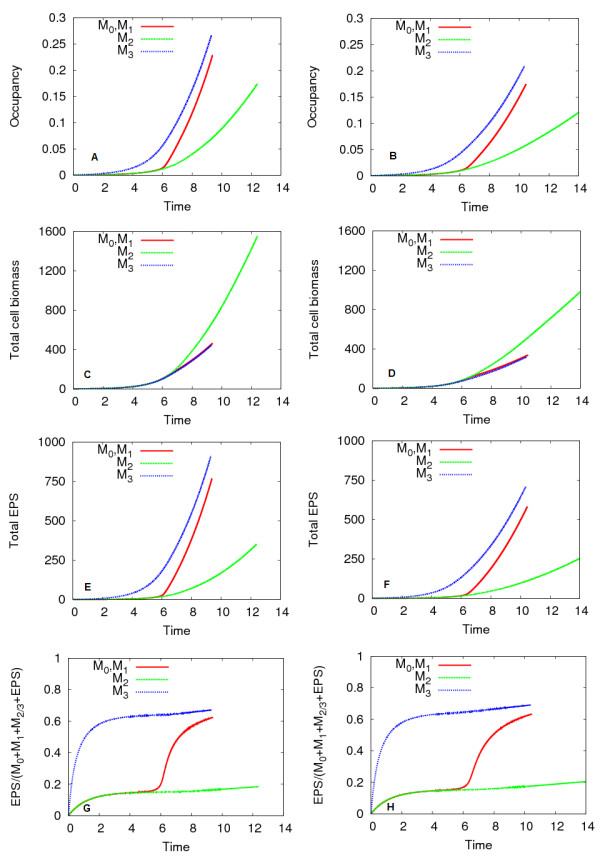
**Spatially averaged results for QS and non-QS biofilms under the high and low nutrient condition**. EPS consumption was excluded. The biofilm occupancy is shown in (A, B), total cell biomass in (C, D), total EPS in (E, F), and fraction of EPS in (G, H) for the high and low nutrient conditions, respectively.

Figure 4 shows the spatially averaged results for these biofilm simulations. The nutrient supply directly influences growth rates, and so the simulations finish earliest for the high nutrient case. The occupancies of the biofilms are shown in Figures 4(a, b); note that occupancy is inclusive of both cells and EPS. The high-EPS producing *M*_3 _non-QS biofilm had the greatest occupancy, followed by the QS biofilm, and the low-EPS producing *M*_2 _non-QS biofilm had the lowest occupancy. In contrast, the *M*_3 _non-QS biofilm had the lowest bacteria cell population, and the *M*_2 _non-QS biofilm had the highest cell population (Figures 4(c, d)). The high values of QS and *M*_3 _non-QS biofilm occupancies are therefore not due to additional bacteria cells, but are due to the presence of EPS produced at the induced rates. This is verified in Figures 4(e, f), which demonstrates the enhanced EPS production by the QS biofilm. The spatial patterns of colony size discussed in the Section "Example simulation of QS controled EPS production in biofilm" are observed in these simulations as well; the upstream colonies experience the most cellular and EPS growth, whereas the cells in nutrient-poor downstream colonies produce less EPS and are slow-growing. These results are consistent under both the high and low nutrient regimes, though the low nutrient regime yielded lower occupancies, cell populations, and total EPS. Because the threshold AHL concentration is surpassed before nutrient limitations occur, induction occurs at the same time in both the high and low nutrient regimes.

The composition of the biofilm over time is shown in Figures 4(g, h). After the initial time period in which EPS production begins, the low-EPS producing non-QS biofilm (*M*_2_) remains composed of less than 20% EPS by mass, whereas the high- EPS producing non-QS biofilm (*M*_3_) is approximately 65% EPS. The QS biofilm switches its composition by mass after induction from predominantly bacteria cells to EPS. In summary, the QS biofilms obtained greater cell populations than *M*_3 _non-QS biofilms, and occupied more volume in the channel than *M*_2 _non-QS biofilms.

The quorum sensing mechanism is used to switch behaviours from the *M*_2 _like mode of low EPS production and faster cell growth to the *M*_3 _like mode of high EPS production at the expense of slower bacterial growth. This transition takes place rapidly after induction occurs and is almost completed after a period of time that is about twice as long as the characteristic time scale of biomass growth. Eventually the entire biofilm behaves like a high-EPS producing *M*_3 _biofilm.

#### Mixed biofilms

**Figure 5:**
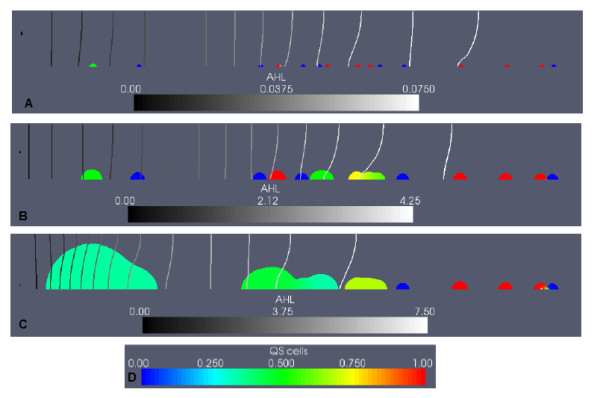
**Simulation of the growth of a *M*_2 _mixed biofilm (*M*_0_, *M*_1_, *M*_2_) under high nutrient conditions**. The process of EPS consumption was excluded. The biofilm is shown at three different time steps throughout the simulation, with contour lines showing the AHL concentrations. Colonies are coloured to represent the fraction of cells which are QS: ((*M*_0 _+ *M*_1_)/(*M*_0_+*M*_1_+*M*_2_)); the legend is given in (D). (A) shows an early development stage of a *M*_2 _mixed biofilm (t = 5.0). In (B), QS and *M*_2 _non-QS colonies are growing and merging (t = 8.0). A late development stage is shown in (C) (t = 11.0).

A series of simulations were performed to simulate the growth of mixed biofilms with a high nutrient supply. Mixed biofilms contain both QS cells and *M*_2 _or *M*_3 _non-QS cells in the channel. An example of growth of a *M*_2 _mixed biofilm is shown in Figure 5. The colour represents the fraction of QS (*M*_0_, *M*_1_) cells in the biofilm, relative to all cells ((*M*_0 _+ *M*_1_)/(*M*_0 _+ *M*_1 _+ *M*_2/3_)). Isolines of AHL concentration are given as well; wherever concentrations are greater than the induction threshold, QS cells have upregulated from *M*_0 _to *M*_1_. Figure 5(a) shows the biofilm at an early development stage. The colonies are approximately equal in size, as carbon supply is sufficient for maximal growth at this time. Because QS induction has not yet occurred, and *C *is not limited, all QS and *M*_2 _non-QS colonies produced EPS equivalently, at the low rate. In Figure 5(b), merging of QS and non-QS colonies is prevalent upstream and in the mid-channel, though colonies at the downstream extent of the biofilm remain exclusively QS or *M*_2 _non-QS. AHL concentrations have surpassed the induction threshold in the mid-channel and downstream, indicating that QS cells in those regions of the domain have upregulated to *M*_1_, and are consuming nutrients at an increased rate. Merging of colonies continues into the late development stage (Figure 5(c)). At the end of the simulation, QS cell populations are slightly higher than *M*_2 _non-QS cell populations. The large, merged, upstream colony experiences the most growth, due to its proximity to the nutrient source, enabling both cell population growth and induced EPS production by upregulated QS cells. Note that in this example, the colony closest to the inflow boundary is a QS colony, and several of the furthest downstream colonies, where nutrient deficiencies are highest, are *M*_2 _non-QS colonies.

**Figure 6:**
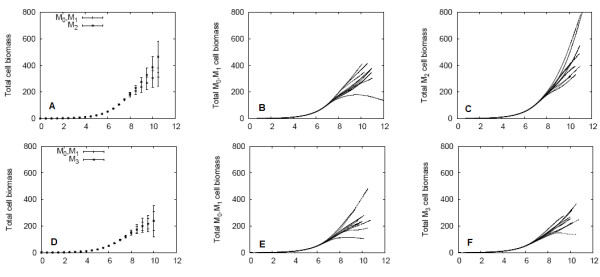
**Biomass of QS and *M*_2 _and *M*_3 _non-QS colonies in mixed biofilms**. High nutrient case, EPS consumption excluded. Figure (A) shows the average biomass and standard deviation for the ten *M*_2_-mixed simulations; and total *M*_0 _+ *M*_1 _(B) and *M*_2 _(C) cell biomass for each individual simulation are shown as well. Similarly, the results for the ten *M*_3_-mixed biofilms are shown in (D)-(F).

Because the resulting final QS and non-QS cell populations may be impacted by the stochastic distribution of cells in the initial inoculation, particularly, whether a QS or non-QS colony is located closest to the nutrient-rich upstream boundary, this *M*_2 _mixed biofilm experiment was repeated for a total of ten simulations, each using a different initial distribution. An additional ten simulations were conducted to test the growth of *M*_3 _mixed biofilms, which include the high-EPS producing *M*_3 _non-QS cells instead of *M*_2 _non-QS cells. The total QS and non-QS biomass averaged over all ten simulations for the *M*_2 _and *M*_3 _biofilms is shown in Figures 6(a, d), and the QS and non-QS cellular biomass for each of the individual simulations are shown in Figures 6(b, c, e, f). It was found that on average, low-EPS producing *M*_2 _non-QS cells outnumbered QS cell populations in the *M*_2 _mixed simulations. The high-EPS producing *M*_3 _non-QS cell populations were approximately equivalent to QS cell populations in the *M*_3 _mixed simulations. In analyzing each of the twenty mixed simulations, it was found that the colony placed most upstream in the random initial inoculation ultimately grew the largest cell population in that simulation, just as was observed in the example *M*_2_-mixed simulation shown in Figure 5. This shows the strong effect of nutrient availability and mass transfer on biofilm structure, which can dominate quorum sensing effects.

In some cases, the upstream colonies utilized so much of the incoming nutrient that cells in the downstream colonies experienced cell death (this is evident by the decreasing curves in the cell biomass plots Figures 6(b, c, e, f)). In accordance with the results of the previous section, populations of high-EPS producing biofilms are lower than populations of low-EPS producing biofilms.

### Simulations with the EPS consumption process

An additional complexity was considered in our biofilm simulations, which accounts for the utilization of EPS by bacteria cells as a secondary source of the carbon nutrient when limitations occur. The experiments of the subsections "Quorum sensing and non-quorum sensing biofilms" and "Mixed biofilms" were repeated, but with consideration of this biological process.

#### Quorum sensing and non-QS biofilms

QS and non-QS biofilms were grown in the channel using the same initial distribution of colonies as the first simulation experiment, under high and low nutrient conditions. The trend discussed there is present here as well: QS biofilms have higher cell populations than *M*_3 _non-QS biofilms, and higher occupancies than *M*_2 _non-QS biofilms. Cell populations were approximately equivalent to those in the biofilms that did not consume EPS, however, occupancy was considerably lower, due to EPS lost through consumption.

When QS biofilms induced, they increased their volume from that of a *M*_2 _non-QS biofilm to match the size of a *M*_3 _non-QS biofilm. These trends are also present under the low nutrient regime, though cell populations and occupancies are lower than when the high nutrient regime was used.

**Figure 7:**
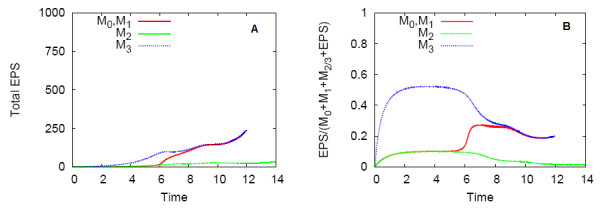
**QS and non-QS biofilms under the high nutrient condition, for the EPS consumption case**. (A) shows the total EPS, and (B) shows the fraction of EPS biomass in the biofilms.

Differences in the biofilm composition were found when biofilms consumed EPS as an additional nutrient source, in comparison to biofilms which do not consume EPS. Figure 7(a) shows the total EPS produced by the biofilms over time for the simulations with a high nutrient supply. In contrast to the non-EPS consuming biofilms in Figure 4, the total net EPS production of these biofilms is much lower, and production is impaired when nutrient deficiencies emerge at approximately *t *= 6.0, when EPS consumption is triggered. Induction of the QS biofilm also occurs at *t *= 6.0, so its total EPS is increased to the levels of the *M*_3 _non-QS biofilm. The fraction of EPS biomass in the biofilm over time for the simulations with a high nutrient supply is shown in Figure 7(b). After the initial time period in which EPS production begins, the low-EPS producing *M*_2 _non-QS biofilm is composed of 10% EPS by mass, whereas the high-EPS producing *M*_3 _non-QS biofilm is about 50% EPS. The QS biofilm switches its composition by mass after induction from predominantly bacteria cells to EPS. When EPS consumption increases, EPS declines to 20% in the *M*_3 _non-QS and QS biofilms, and to almost zero in the *M*_2 _non-QS biofilm. Biofilms that use EPS as a nutrient source are predominantly composed of cellular biomass, or entirely by cellular biomass, in extreme nutrient deficiencies.

**Figure 8:**
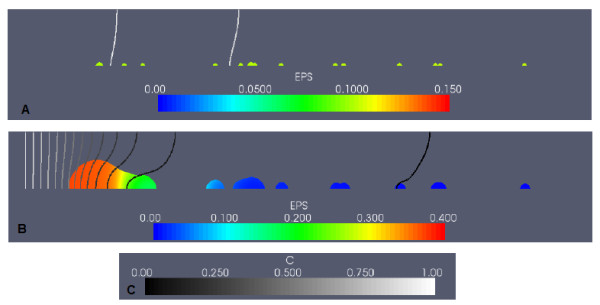
**An example of a QS biofilm under high nutrient conditions, EPS consumption case**. The composition of the biofilm by EPS biomass is shown (A) before and (B) after induction. The carbon concentration scale is given in (C).

However, the most notable difference in biofilms that consumed EPS was in the biofilm composition. The EPS consumption process promotes more pronounced spatial differences in biofilm composition. In Figure 8(a), a QS biofilm with the same initial inoculation of Figure 2 is shown at an early development stage, when nutrient supply is abundant, and composition is uniformly 10% EPS. In Figure 8(b), induction has occurred, and the large, merged upstream colony has experienced growth and enhanced EPS production by upregulated *M*_1 _cells. Only the most upstream portion of this colony contains both cells and EPS. Here, as indicated by the carbon isolines, upregulated cells have access to the nutrients required to produce EPS at the induced rate, and therefore do not need to consume local EPS for additional nutrients. Elsewhere, the cells are starved of nutrients and are forced to consume their entire EPS supply.

After QS induction occurs, the biofilm undergoes a transition from a low EPS producing *M*_2 _like state to a state that is essentially entirely *M*_3 _like, i.e. it becomes a fast EPS producer at the expense of slower bacterial growth. After this transition is complete, within a time period that compares to the characteristic timescale of biomass growth, no remainders of the down-regulated past are evident. This corresponds to the observations of the previous sections.

#### Mixed biofilms

**Figure 9:**
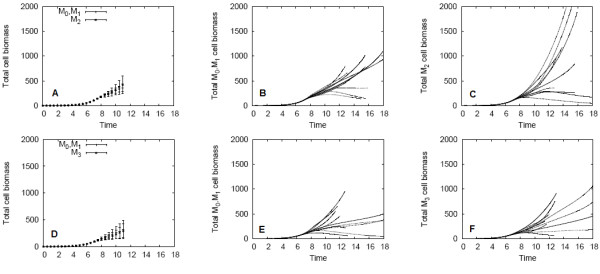
**Biomass of QS and non-QS colonies in mixed biofilms**. High nutrient case, EPS consumption included. Figure (A) shows the average biomass and standard deviation for the ten *M*_2_-mixed simulations; and total *M*_0 _+ *M*_1 _(B) and *M*_2 _(C) cell biomass for each individual simulation are shown as well. Similarly, the results for the ten *M*_3_-mixed biofilms are shown in (D)-(F).

Twenty additional simulations of mixed biofilms under the high nutrient condition were performed (ten *M*_2_-mixed and ten *M*_3_-mixed biofilms), each using a different initial inoculation, with the inclusion of the EPS consumption process. Figure 9(a, d) shows the average biomass of the QS and non-QS colonies, as well as the total cell biomass in time for every simulation in Figures 9(b, c, e, f). In comparison to the mixed biofilms that excluded EPS consumption, a higher variance in final cell populations was observed. This is in part due to an increased occurrence of cell populations either remaining constant or declining in time. Again, the average QS and non-QS cell populations are not higher than the mixed biofilms which excluded EPS consumption. Similar to the previous mixed simulations, biofilms with low-EPS producing *M*_2 _non-QS cells obtained higher cell populations than biofilms with high-EPS producing *M*_3 _non-QS cells. In every case, the colony located closest to the nutrient source experienced the most growth in the particular simulation. Decline of cell populations, observed in some of the simulations, is attributed to a high distribution of those colonies in the nutrient-poor mid-channel and downstream regions. Spatial gradients in biofilm composition by EPS, as discussed in subsection "Quorum sensing and non-QS biofilms" (in "Simulations with the EPS consumption process"), were also prevalent, resulting in biofilms with little to no EPS in the downstream colonies.

### Effect of random colony placement in mixed biofilms

**Figure 10:**
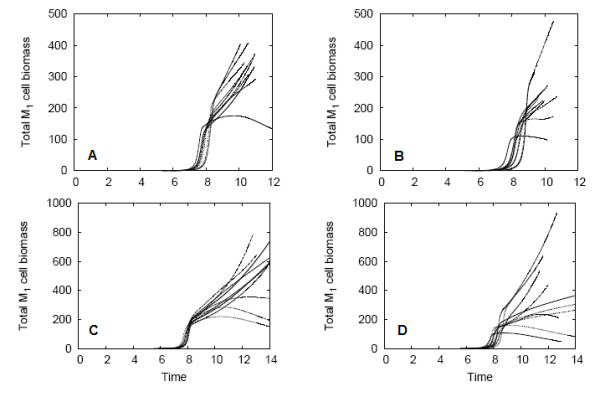
***M*_1 _biomass in the mixed biofilm simulations**. (A) and (C) are *M*_2_-mixed biofilms, without and with EPS consumption. (B) and (D) are *M*_3 _mixed biofilms, with and without EPS consumption.

In subsections "Mixed Biofilms", in both "Simulations without the EPS consumption process" and "Simulations with the EPS consumption process", variations in the total QS and non-QS biomass were found for the *M*_2_- and *M*_3_-mixed biofilms. To investigate whether these variations are attributed to nutrient limitations or to QS, the *M*_1 _biomass was studied for all forty mixed biofilm simulations, plotted in Figure 10.

The positive feedback feature of QS systems leads to a very rapid upregulation of QS cells in the biofilm -- after induction occurs, *M*_1 _cell populations quickly rise. The switching time represents this upregulation period. In Figures 10(a, b), the *M*_2_- and *M*_3_-mixed biofilms that exclude EPS consumption have a switching time between 7.5 and 8.5. The *M*_2 _and *M*_3 _mixed biofilms that consume EPS (Figures 10(c, d)) switch between 7.5 and 8.0. Though the variation in switching time is small, we can conclude that the random initial placement of colonies does have a small effect of the time at which QS induction occurs. After induction, nutrient limitations arise, and the variability in total *M*_1 _cell biomass increases. Nutrient shortages occur not only because the biofilm is larger, but also because the upregulated *M*_1 _cells have a higher consumption rate. In each simulation, nutrient limitations cause *M*_1 _cell growth rates to decrease, and in some cases, the cell populations remain constant or even decline.

## Discussion

The first question that motivated our study was: *how does QS-regulated EPS production impact the growing biofilm? In particular, what does the biofilm look like over time, with respect to distribution and composition of cells and EPS? *The answers to these questions were obtained by studying biofilm composition and the upstream clogging effect.

It was found that colony growth was so greatly enhanced with high-EPS producing non-QS biofilms, and in induced QS biofilms, that these biofilms rapidly fill the channel with biomass. When high-EPS producing cells are located in a region where nutrient supply is abundant, these cells have access to the nutrients required for enhanced EPS production. If these cells are located in a region of nutrient scarcity, induced EPS production reactions cannot occur, and so colonies cannot undergo enhanced expansion. In all the simulations, spatial gradients of biofilm size were prevalent -- large, merged upstream colonies have full access to available nutrients, grow and fill the channel, and cause the downstream colonies to remain small. At low EPS production rates, the biofilm is composed by majority of cells, at high EPS production rates, the biofilm is predominantly EPS. When QS biofilms induce, they switch their proportions of cellular and EPS biomass. The composition differs greatly if the EPS consumption process is modelled. In regions of nutrient deficiencies, EPS may be completely consumed by the bacteria cells, to the extent that the biofilm consists only of cells (generally in the mid- to downstream region), and only the portion of the biofilm closest to the inflow boundary contains a protective layer of EPS.

We were also interested in investigating, *is it beneficial for the biofilm to regulate EPS production using a QS mechanism? *Several factors are considered in determining whether one biofilm was more successful than another, including occupancy and total cell biomass. High-EPS producing biofilms have higher occupancies than low-EPS producing biofilms, as a result of increased levels of EPS. They may furthermore benefit, for example, by protection from environmental hazards, such as detachment, antibiotics or grazers. Low-EPS producing non-QS cells which have merged with upregulated QS colonies (in mixed simulations) therefore experience benefits from the thick EPS layer as well. If EPS consumption is occurring, non-QS cells in mixed colonies also benefit by consuming the additional nutrients produced.

However, in all simulations, low-EPS producing cells on average outnumbered the QS cells. This occurred in comparing QS with low EPS-producing non-QS biofilms, and mixed biofilms, with and without the EPS consumption process. These findings show that EPS production does not provide a benefit to the biofilm in regards to achieving a high cell population, which is one potential objective of the bacteria cells residing in a biofilm. To produce EPS at the induced rate, bacteria cells have a high nutrient demand, and if this demand is not met, colony growth cannot occur. Another cause of low populations of high-EPS producing cells is that these expanding colonies, with high proportions of EPS, quickly clog the channel. In contrast, low-EPS producing non-QS biofilm colonies take much longer to grow to the point that the channel is clogged, but the biofilm is composed primarily of cells.

In the mixed biofilm simulations, success of QS or non-QS populations was determined by proximity to the nutrient source, largely an effect of the random initial distribution. In a mixed environment, clogging of the channel by upstream colonies may prevent downstream colonies from receiving nutrients, in some cases causing their populations to suffer declines. Resource requirements are considerably higher for high-EPS producing cells. If an adequate nutrient supply can be secured, either by locating a colony near the nutrient source or utilizing EPS as a secondary food source, then it is possible for QS cell populations to be greater than low-EPS producing cell populations.

Under the investigated conditions, we found that overall, QS-regulated EPS production rarely provides a benefit to a biofilm with the objective of achieving a high cell population, in comparison to biofilms with low EPS-producing non-QS cells. However, maximizing offspring generation is not necessarily the best strategy under all conditions. EPS production would be beneficial if the objective of the bacteria cells in the biofilm is to clog the channel. A QS biofilm colony located near the nutrient source may use QS to increase its volume, clog the channel, and secure its supply of nutrients while starving downstream colonies, and potentially force the downstream colony to deplete their EPS supply. This is a competitive advantage for a colony, whether it is located in a QS biofilm (indicating intra-species competition), or in a mixed biofilm (inter-species competition). In any space-limited environment, such as fine sediment or small vessels in higher organisms, channel clogging may be considered a beneficial strategy for bacteria cells to use. In the study [51], it was shown that biofilms developing in plants stems used QS to clog the plant vessel used for water and nutrient transportation, the so-called xylem, securing nutrients for themselves. Individual colonies in patchy biofilm communities have an antagonistic interaction (some colonies benefit at the expense of others) through utilization of an exploitative competition strategy - the upstream colonies reduce nutrient resources in the environment, depleting the resource required by the downstream colonies. If cells wish to outcompete another cell population in a biofilm, of their own species or a different species, (QS-controlled) EPS production can provide an advantage. The drawback is that nutrient supply must be abundant, as high-EPS producing cells have a high nutrient demand.

Downstream cells are upregulated (partly) by AHL produced by upstream colonies. However, it is not obvious how upstream colonies could contribute to a potential benefit (e.g. protection) for downstream cells through EPS production. In this sense, downstream cells seem to be fooled into a premature EPS production, which they might not even be able to realize due to nutrient depletion.

The results showed that on a population level, high-EPS producing biofilms suffered lower cell populations. However, the spatial distributions in the 2D visualizations demonstrated that for individual colonies, clogging the channel with biomass may be a beneficial strategy. Individual cells within microcolonies are genetically related, and have the ultimate objective of maintaining their own genes and reproducing in the future. To optimize survival of their genes in the colonies, and ensure survival of their offspring, bacteria cells would be interested in suppressing other colonies. In the event of a structural reorganization or detachment/reattachment, the upstream colony has an advantage, in that their genetically similar offspring will continue to succeed.

The nutrient supply, random placement of colonies, and QS had various impacts on the resulting biofilms. QS and high-EPS producing non-QS biofilms had almost equivalent total cell biomass under both the high and low nutrient regimes, indicating that total cell population at the end of the simulation is predominantly controlled by nutrients and mass transfer in the aqueous phase, and not quorum sensing, which is also supported by the experimental findings of [63]. It was found that the random placement of colonies in the initial inoculation had a small impact on when induction occurred. Nutrient limitations succeeded induction, and led to greater variability in total cell biomass. The impact of QS was most prominent in regards to biofilm occupancy and composition. QS induction occurs very rapidly, and likewise, the QS biofilms were able to rapidly increase their occupancy and change their composition from that of a low EPS-producing to high EPS-producing non-QS biofilm.

We argued that EPS production benefits cells under certain conditions. The question arises of why EPS production would be associated with quorum sensing, that is, why would cells wait until upregulation to produce EPS at higher rates, versus always producing EPS at enhanced levels? A minimum thickness of an EPS layer is required for effective protection of cells from a hazard, such as antibiotics and washout. If a cell population is small, much energy would be expended to produce a layer of EPS which may not provide adequate protection. Coupling EPS production with quorum sensing ensures that a sufficient colony size is obtained such that the cost to produce EPS is returned by the benefits of a protective layer. QS may then be considered a mechanism for switching to a mode of high-EPS production once a certain number of cells is present, ensuring the bacteria can protect themselves efficiently. Rather than existing in a permanently upregulated state, which requires additional nutrients, QS biofilms can quickly switch to the upregulated state and increase their size when necessary.

## Conclusions and further work

In this study, we developed a mathematical model of QS in biofilms which incorporated an effect of QS on biofilm growth: upregulated cells produce EPS at an increased rate. Through simulations, we investigated QS, non-QS, and mixed biofilms under various nutrient regimes and the biological process of EPS consumption. Our main results are:

• QS-induced EPS production allows a biofilm to switch rapidly from an initial colonization stage, in which the focus lies on rapid cell production, to a stage in which the focus lies on producing large amounts of EPS as protection against environmental threats such as grazers, mechanical washout, biocides, and transient periods of nutrient limitation.

• low-EPS producing biofilms generally obtained higher cell populations, and high-EPS producing biofilms obtained greater occupancies. The QS biofilms rapidly changed from a state of cell growth to a state of EPS production; in other words, quorum sensing is used as a signal for the biofilms to switch from a colonization mode to a protection mode.

• high-EPS producing biofilm colonies rapidly filled their space-limited environment with biomass, and consisted of high proportions of EPS biomass. When EPS is consumed, however, extreme nutrient deficiencies lead biofilms to deplete their EPS supply, resulting in biofilms comprised purely of cells, with little to no EPS present.

• a biofilm will benefit from using quorum sensing-induced EPS production if bacteria cells have the objective of acquiring a thick, protective layer of EPS, or if they wish to clog their environment with biomass as a means of securing nutrient supply and outcompeting other colonies in the channel, of their own or a different species.

Limitations of our model and simulation setup include the narrow simulation space. We chose to model biofilm growth in narrow channels, and because high-EPS producing biofilm colonies expand rapidly, the observation period of QS induced biofilms is brief. Other experimental setups that allow for long-term biofilm development could be of interest; our model is intended to describe only the initial stages of biofilm growth in a reactor system representative of space-restricted environments with fluid flow, such as soil pores.

We used our model to observe connections between biofilm growth, quorum sensing, and EPS production. Our study could be extended to test biofilms that use quorum-sensing induced EPS production under the scenarios which we speculated biofilms would benefit from high levels of EPS, for example, when grazers or antibiotics are present. Future work in biofilm and quorum sensing modelling is required to continue to investigate how biofilms respond to quorum sensing induction.

## Competing interests

The authors declare that they have no competing interests.

## Authors contributions

This study was conducted as part of the M.Sc. thesis research of MRF at the University of Guelph, and on research visits to the HelmholtzCenter Munich. HJE was the thesis supervisor and guided the biofilm modelling aspects, CK and BAH guided the quorum sensing modelling aspects and the ecological interpretation of the results. All authors contributed to the writing of the article, and read and approved the final manuscript.

## Acknowledgements

This study was supported in parts by the Advanced Foods and Materials Network, a Network of Centers of Excellence (NCE) and the National Science and Engineering Research Council (NSERC). The computing equipment used for this study was provided by the Canada Foundation for Innovation (CFI) with a grant in the Leaders Opportunity Funding program, awarded to HJE. We thank the technical staff of the Shared Hierarchical Academic Research Network (SHARCNET), for their technical support.
